# The vaginal microbiome and sexually transmitted infections are interlinked: Consequences for treatment and prevention

**DOI:** 10.1371/journal.pmed.1002478

**Published:** 2017-12-27

**Authors:** Janneke H. H. M. van de Wijgert

**Affiliations:** 1 Julius Center for Health Sciences and Primary Care, University Medical Center Utrecht, Utrecht, The Netherlands; 2 Institute of Infection and Global Health, University of Liverpool, Liverpool, United Kingdom

## Abstract

In a Perspective for our Collection on STI research, Janneke van de Wijgert discusses the latest on how the vaginal microbiota predisposes women to acquisition of STIs and discusses future potential for clinical intervention.

The increased availability of high-throughput molecular testing since the turn of the century has revealed a more detailed picture of organisms that may be present in the vagina than was possible when diagnosis depended on microscopy, culture, and—in the case of sexually transmitted infection (STI) pathogens—polymerase chain reaction (PCR). While past research focused individually on STIs, bacterial vaginosis (BV), vulvovaginal candidiasis (VVC), and vaginal carriage of *Streptococcus agalactiae* as a cause of neonatal disease, new understandings of the interrelationships among vaginal organisms, and their effects on the cervicovaginal mucosal barrier and immune system, have advanced understanding of relationships between the vaginal microbiome and a variety of adverse outcomes, including HIV acquisition, pelvic inflammatory disease, miscarriage, preterm birth, and invasive maternal and neonatal infections [1–4].

## Healthy and dysbiotic vaginal environments

While a cervicovaginal mucosa covered with lactobacilli is still considered the optimal environment, molecular studies have shown that not all lactobacilli are equal [1]. *Lactobacillus crispatus* only occasionally co-occurs with organisms other than lactobacilli, has been associated with an anti-inflammatory cervicovaginal immune profile, and seems to protect women from developing anaerobic dysbiosis and from the above-mentioned adverse outcomes [1–5]. In contrast, *L*. *iners* does not seem to protect women from developing anaerobic dysbiosis and often co-occurs with BV-associated anaerobes, pathobionts (streptococci, staphylococci, or *Enterobacteriaceae*), or pathogens [1–6]. A vaginal microbiome dominated by *L*. *iners* is, however, not associated with a proinflammatory profile, and data on whether it increases the risk of adverse outcomes are conflicting. Vaginal microbiomes with a high relative abundance of the other vaginal lactobacilli are much less prevalent and less well studied [1,7].

Vaginal dysbiosis is often defined as a prolonged deviation from a low-diversity, lactobacilli-dominated vaginal microbiome. Molecular studies have identified different types of vaginal dysbiosis [1,7]. The most common type is high-diversity anaerobic dysbiosis, almost always including *Gardnerella vaginalis* and *Atopobium vaginae* as well as multiple other anaerobes, with or without a low relative abundance of *L*. *iners*. Low-diversity anaerobic dysbiosis, characterized by *G*. *vaginalis* or *A*. *vaginae* domination, also occurs, albeit less commonly. Studies employing multiple methods of vaginal microbiome characterization have shown high correlations between BV by Gram stain Nugent scoring and anaerobic dysbiosis (high and low diversity combined) [1]. Another type of vaginal dysbiosis that is likely important from a clinical point of view is a high relative abundance of pathobionts, also referred to as pathobiont carriage. Anaerobic dysbiosis and pathobiont carriage have been associated with proinflammatory immune profiles and with the above-mentioned adverse outcomes, and anaerobic dysbiosis has also been associated with cervicomucosal barrier disruption [3]. The roles of *Bifidobacteriaceae* (other than *G*. *vaginalis*) and *Corynebacterium* in the vaginal microbiome have not yet been studied, and domination by these bacteria is rare [1,7].

**Fig 1:**
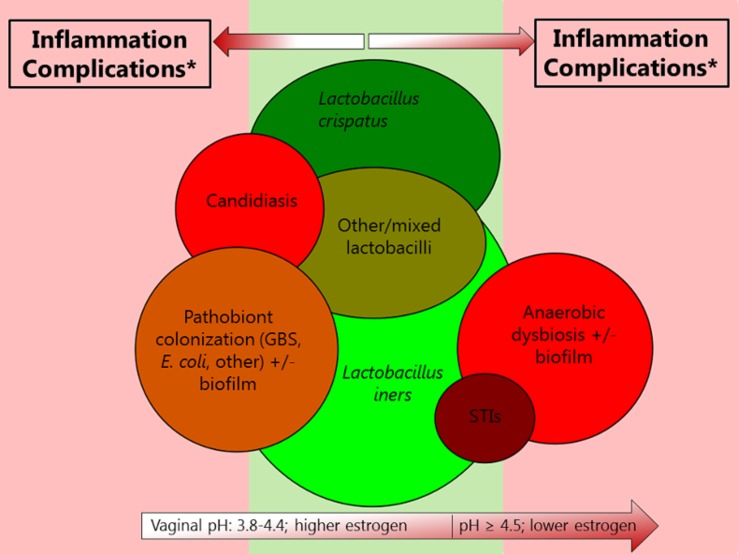
Visualization of interrelationships among various urogenital conditions involving micro-organisms. Green colors indicate desirable conditions, and red colors indicate undesirable conditions. In both cases, the darker the color, the more desirable or undesirable the condition, respectively. The size of the circles is relative to the size of the respective epidemics, but only very roughly. The STI circle does not include viral STIs. The circles on the far left and far right appear as if they do not overlap because the image is two dimensional, but they do overlap somewhat. It is important to note that few studies on the associations between urogenital conditions and host responses or adverse outcomes (which determine whether a condition is desirable or undesirable) have been holistic. For example, many studies only employ 16S ribosomal RNA sequencing of the vaginal microbiota, but this does not cover fungi, protozoa, and viruses and does not reliably identify *Chlamydia trachomatis* and *Neisseria gonorrhoeae*. Abbreviations: GBS, Group B streptococcus; STI, sexually transmitted infection. * Complications include HIV acquisition, pelvic inflammatory disease, adverse pregnancy outcomes, and maternal and neonatal infections.

Many epidemiological studies have found that BV, VVC, vaginal pathobiont carriage, and STIs are interrelated and that many of the associations are bidirectional [8,9]. The interrelationships could be explained by both behavioral and biological factors. First, many of these conditions share risk factors related to sexual transmission. While BV, VVC, and vaginal pathobiont carriage were never considered STIs and can indeed occur in the absence of sexual activity, it is now clear that sexual transmission of the implicated organisms does play a role, especially in sex with uncircumcised male partners [2,10]. Second, most dysbiosis types and VVC cause mucosal barrier disruption, which decreases the ability of mucus and vaginal secretions to trap or inactivate pathogens and creates epithelial portals of entry, and cervicovaginal inflammation, which increases the concentration of target cells for HIV at the mucosal sites where HIV exposure takes place [3]. Interestingly, STIs and anaerobic dysbiosis often overlap, but VVC seems to occur more often in the presence of lactobacilli-domination than in the presence of anaerobic dysbiosis (Fig 1) [8]. A recent study showed positive associations between vaginal *S*. *agalactiae* carriage and vaginal *Escherichia coli* and *Candida albicans* carriage, but a negative association with anaerobic dysbiosis [9]. Many explanations for these associations have been hypothesized, such as vaginal pH (*C*. *albicans*, *S*. *agalactiae*, and *E*. *coli* are not inhibited by the low vaginal pH produced by lactobacilli), competition between micro-organisms for nutrients, microbial defense mechanisms against one another, biofilms that include some micro-organisms but not others, and attachment of some bacteria to *Candida* hyphae. Further in-depth characterization of these mechanisms is important because they may lead to new targets for drug development and an increased understanding of how intervening in one pathway might influence other pathways.

## Implications and challenges

While first-line treatment of BV with oral or vaginal metronidazole or clindamycin is typically efficacious in the short term (as defined by Nugent or Amsel criteria), recurrence rates are high [10,11]. Clinical studies have shown that BV recurrence rates can be reduced by longer duration and/or prophylactic use of first-line antibiotics, by (estrogen-containing) hormonal contraception, and by circumcision of male sexual partners, but not by adding other antibiotics (azithromycin or moxifloxacin) to first-line antibiotics or by metronidazole/clindamycin treatment of male sexual partners [11]. Some argue that recurrence is particularly likely when a mucosal biofilm is present. In vitro and in vivo studies have shown that such a biofilm is damaged and suppressed by metronidazole but not completely eliminated [12]. The interrelationships between various urogenital conditions also pose challenges. For example, treatment of anaerobic dysbiosis often leads to VVC [13]. Treatments might be more efficacious in the longer term when they specifically target dysbiosis-associated anaerobes or pathobionts while sparing lactobacilli and are combined with biofilm disrupting agents, systemic or topical estrogen, and/or *Lactobacillus*-containing vaginal pro- or synbiotics. Estrogen-containing hormonal contraception, and *Lactobacillus*-containing vaginal pro- or synbiotics if found to be clinically effective, could also be implemented for routine use on a larger scale to prevent vaginal dysbiosis in women at risk.

While current knowledge suggests that maintaining lactobacilli-dominant, inflammation-free vaginal environments could advance the prevention of HIV, STIs, and adverse outcomes, this would not be an easy task, and many research questions remain. At the moment, the vaginal health of women is seldom routinely checked, even in pregnancy. HIV/STI screening programs targeting at-risk populations do exist, but otherwise, only women who seek medical care for urogenital symptoms are likely to be evaluated and treated, often in the absence of any diagnostic laboratory testing. Further, there is ample evidence that presumptive and syndromic management of urogenital conditions in the absence of any diagnostic testing have low sensitivity and specificity compared to diagnostic testing followed by treatment [14]. Even if diagnostic testing were to be introduced in order to optimize vaginal health and minimize complications, we currently do not know which women to target, when intervention would be required (i.e., which relative abundances or concentrations of *G*. *vaginalis*, *A*. *vaginae*, and pathobionts or which levels of cervicovaginal inflammation should be considered harmful), and which interventions, or combinations of interventions, would be optimal. However, the progress made in recent years has made it possible to start contemplating these issues and work towards solutions.

## Abbreviations

BV: bacterial vaginosis
PCR: polymerase chain reaction
STI: sexually transmitted infection
VVC: vulvovaginal candidiasis
